# A Hybrid Chitosan–BaTiO_3_ Composite-Based Flexible and Self-Powered Pressure Sensor for Wearable Healthcare Applications

**DOI:** 10.1021/acsaenm.6c00330

**Published:** 2026-06-17

**Authors:** Zhao Wang, Bhavani Prasad Yalagala, Jungang Zhang, Zixuan He, Hadi Heidari, Andrew Feeney

**Affiliations:** † Centre for Medical and Industrial Ultrasonics, James Watt School of Engineering, 3526 University of Glasgow, University Avenue, Glasgow G12 8QQ, U.K.; ‡ Microelectronics Laboratory, James Watt School of Engineering, University of Glasgow, University Avenue, Glasgow G12 8QQ, U.K.; § Materials and Manufacturing Research Cluster, James Watt School of Engineering, University of Glasgow, University Avenue, Glasgow G12 8QQ, U.K.

**Keywords:** pressure sensor, prosthetics, self-powered, biopolymer, chitosan–BaTiO_3_, piezoelectric, organic−inorganic, wearable.

## Abstract

Wearable pressure sensors are key to many applications, such as those for Internet of Things (IoT), implantables in healthcare, soft robotics, insoles, and prosthetics, where the demands for flexible, self-powered, and highly sensitive pressure sensors are significantly growing. These requirements necessitate the development of flexible and high-performance piezoelectric materials, where current embodiments can be limited, requiring compromises between properties such as sensitivity, output voltage, biocompatibility, and response time. Here, a biopolymer composite-based piezoelectric material using chitosan-BaTiO_3_ nanoparticles (NPs) is produced, showing significant promise for wearable healthcare pressure sensors. To demonstrate this, a piezoelectric-based dynamic pressure sensor and its array (5 × 5) are fabricated using a hybrid composite material by varying different concentrations of BaTiO_3_ NPs (5, 10, 15 wt %). The sensors demonstrate relatively high pressure and frequency sensitivities for three different concentrations of the hybrid composite (5, 10, 15 wt %), constituting 105 mV/kPa, 233 mV/kPa, 276 mV/kPa; and 448.57 mV/Hz, 1703 mV/Hz, 2564 mV/Hz, respectively. The results show that the sensitivity increases linearly with an increase in the BaTiO_3_ NP concentration, and the 15 wt % shows a maximum of 11-fold increase in the pressure and frequency sensitivities compared to pure chitosan. The mechanical flexibility of the pressure sensor is validated for 10^4^ cycles and demonstrates excellent long-term stability. This class of hybrid organic–inorganic composite-based pressure sensor will be a pathway toward highly flexible, sensitive, and biocompatible wearable electronic devices for healthcare applications.

## Graphical abstract

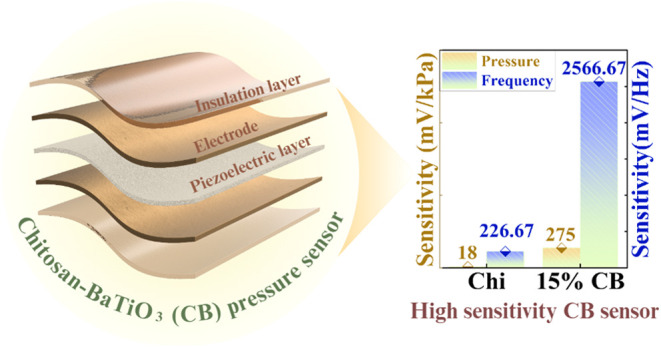

## Introduction

The rapid development of wearable electronic devices, wireless transmission in recent years has significantly increased activity toward the development of flexible and self-powered pressure sensors.^1,2^ The application scope is extensive, particularly in wearable electronics, such as smart gloves and insoles. For example, integrated pressure-detecting insoles can be critical for monitoring and assessing human gait performance, facilitating comprehensive surveillance of walking posture and enabling clinicians to perform clinical diagnosis and treatment of gait-related disorders.^3,4^ Various types of sensors have been employed for pressure detection in insoles, including piezoresistive, capacitive, piezoelectric, and triboelectric.^5–8^ Piezoelectric and triboelectric sensors offer significant advantages because they convert mechanical motion into electrical signals, enabling self-powered operation without external power sources. Traditional inorganic piezoelectric materials such as lead zirconate titanate (PZT) provide a high piezoelectric coupling coefficient and stable pressure sensitivity. However, their environmental toxicity and limited biocompatibility restrict their suitability for wearable electronics. Although alternative materials such as barium titanate (BaTiO_3_) provide nontoxic options, they are rarely used in flexible and stretchable devices due to their rigidity, brittleness, stiffness, and bulky nature. To address these limitations, organic piezoelectric materials such as polyvinylidene fluoride (PVDF), poly­(D-lactic acid) (PDLA), polylactic acid (PLA), parylene C, poly­(vinylidene fluoride-trifluoroethylene) (P­(VDF-TrFE)), gelatin, and polyamides (PA) have been explored for the fabrication of flexible pressure sensors.^9–13^ However, the main limiter of these materials is that they typically exhibit significantly lower piezoelectric coupling coefficients compared to those of inorganic piezoelectric materials. In order to satisfy the demands of the practical applications, a commonly adopted method is to change the composition of the materials to enhance the piezoelectric properties, such as doping inorganic nanoparticles (NPs, such as BaTiO_3_ or ZnO) into organic polymers.

In our current work, chitosan was selected as a biosourced piezoelectric material due to its significant advantages, including its nontoxicity, biocompatibility, biodegradability, natural abundance and sustainability. BaTiO_3_ NPs were chosen as the additive material for embedding into chitosan, as BaTiO_3_ is regarded as one of the most promising lead-free ferroelectric piezoelectric materials with a high piezoelectric coupling coefficient and compliance with environmental and biosafety requirements.^14^ The biocompatibility of chitosan-based sensing materials has been reported by previous in vitro studies. Yalagala et al. evaluated the biocompatibility of chitosan films by culturing mouse aortic smooth muscle cells (MASMCs) on chitosan films, with control culture plates without films used as the reference. Under standard cell culture conditions (37 °C, 5% CO_2_, 95% humidity, 24 h), the cell viability on the chitosan films was above 95%, with no significant difference compared to the control group, indicating no obvious cytotoxicity and good biocompatibility of chitosan.^15,16^ Prokhorov et al. further investigated the biocompatibility of chitosan/barium titanate (CB) nanocomposites using human foreskin fibroblast (HF) cells cultured in DMEM medium under the same conditions. While CB films with BTO concentrations above 10 wt % exhibited a cell damage rate exceeding 12.7%, indicating content-dependent biocompatibility, hydroxylated BTO nanoparticles did not adversely affect HF cell viability in CB composite films over the concentration range of 1 to 30 wt %.^17^ The resulting CB piezoelectric films combine the high piezoelectric response of BaTiO_3_ with the flexibility, biocompatibility, and nontoxic character of chitosan, showing the potential for wearable healthcare applications. Although some current research also involves chitosan-based or BaTiO_3_-enhanced hybrids, studies on the application of CB for self-powered pressure sensors remain incomplete. Murugan et.al. have explored chitosan loaded with ∼120 nm BaTiO_3_ particles at various concentrations for detecting 80 Hz, 1 g-level dynamic vibrations, investigating sensor sensitivity across different BaTiO_3_ concentrations. However, no further research was conducted on flexibility, piezoelectric properties, self-powering capabilities, or biocompatibility.^18^ Similarly, Pongampai et al. have incorporated BaTiO_3_ nanorods into chitosan films to enhance output performance. However, this study analyzed the coupling of piezoelectricity and triboelectricity without separately investigating the piezoelectric properties.^19^ Next, a similar study from Prokhorov et.al. demonstrated the piezoelectric properties of CB piezopolymers, yet the focus of the study centered on biological responses, demonstrating favorable biocompatibility and promotion of cell growth, without exploring its characteristics as a pressure sensor.^20^ Indeed, extensive research indicates BaTiO_3_ serves as an excellent filler to enhance the performance of organic flexible piezoelectric materials. However, most current studies focus on earlier-discovered organic flexible materials such as PVDF,^21–25^ PVC polymer,^26^ PVDF–TrFE,^27^ and PDMS.^28^ For example, Lennox et al. utilized a Ba0.9Sr0.1TiO_3_/PVDF polymer composite combining piezoelectric coefficient and flexibility to create a self-powered pressure sensor capable of detecting plantar pressure distribution and performing real-time gait analysis.^29^ Few investigations have explored the integration properties of naturally derived biocompatible chitosan with BaTiO_3_ materials. This paper therefore focuses on investigating the piezoelectric properties, output voltage, biocompatibility, flexibility, and stability of self-powered pressure sensors fabricated from CB composites.

The resulting CB film was utilized to develop a novel biocompatible and self-powered dynamic pressure sensor for insole applications, as illustrated in Figure 1. The insole integrates wireless Bluetooth technology via nRF52840, feather, enabling real-time monitoring of gait information and data transmission to mobile devices. The effective wireless transmission distance ranges from 5 to 10 m, with a maximum signal transfer delay of 1 ms and more details were already discussed elsewhere.^30^ The sensor array consumes approximately 7 mA during active wireless data transmission. The CB pressure sensor combines the advantages of organic chitosan and inorganic BaTiO_3_ NPs forming the active piezoelectric layer and in a sandwich structure between two copper electrode layers, where the NPs were formulated in different concentrations of 5, 10, and 15 wt %. The composite CB film exhibits relatively high sensitivities of 276 mV/kPa and 2564 mV/Hz, which demonstrates a significant enhancement in both pressure and frequency sensitivities, nearly 11-fold (both), compared to those for pure chitosan film. Furthermore, the piezoelectric coefficient (*d*_33_) shows an approximately 2-fold increase, with measured values of 7 pC/N (pure chitosan film) and 13 pC/N (15 wt % CB film), attributed to the presence of BaTiO_3_ NPs. In this research, the morphology and crystalline structure of BaTiO_3_ NPs synthesized in chitosan substrates with different concentrations were investigated by scanning electron microscopy (SEM) and X-ray diffraction (XRD), while Fourier-transform infrared spectroscopy (FTIR) was used to characterize the chemical interactions between chitosan and BaTiO_3_ NPs. Subsequently, the output voltage response and sensitivity under various mechanical pressures and frequencies of the fabricated CB pressure sensors were systematically evaluated and compared. The developed CB pressure sensor demonstrates considerable promise for advanced wearable sensing applications, particularly in fields such as human–machine interfaces, telemedicine, sports performance monitoring, and personalized healthcare.

**Figure 1:**
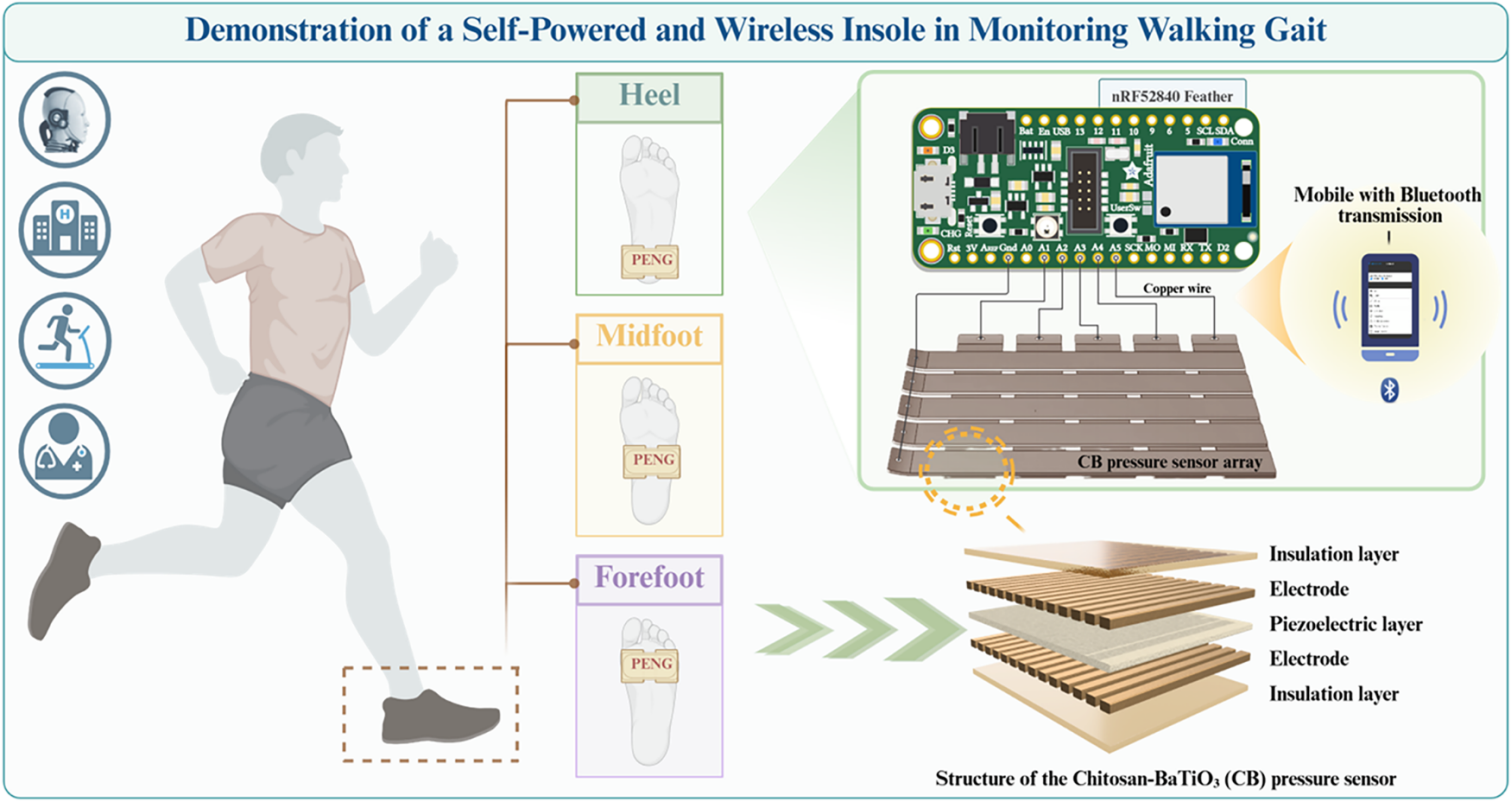
Schematic illustration of a wearable, self-powered wireless insole for gait monitoring. The insole integrates piezoelectric sensors at the heel, midfoot, and forefoot, transmitting data via Bluetooth (nRF52840 Feather) to a mobile device. The insets show the structure of a Chitosan–BaTiO_3_ pressure sensors, with potential applications highlighted, including human–machine interaction, telemedicine, sports monitoring, and healthcare. Created in BioRender. Wang, Z. (2026) https://BioRender.com/nj4thhj.

## Materials and Methods

### Materials

The chemicals and materials used included chitosan (low molecular weight, 9012–76–4, MFCD00161512), acetic acid (glacial, ReagentPlus, ≥99%, 64–19–7, MFCD00036152), and BaTiO_3_ (99.9% trace metals basis, 12047–27–7, MFCD00003447). All materials were obtained from Merck Life Science (Dorset, SP, UK) and were used without further purification.

### Preparation of Chitosan-BaTiO_3_ (CB) Film

Chitosan was prepared using a 2 wt % solution in acetic acid and DI water, where a more detailed preparation methodology is reported in our previous works and as shown in Figure 2.^14^ For the composite films, BaTiO_3_ NPs (1, 2, and 3 g) were separately incorporated into three 100 mL aliquots of chitosan solution to achieve final BaTiO_3_ concentrations of 5, 10, and 15 wt %, respectively. Each mixed solution was subjected to magnetic stirring, ultrasonication, and vacuum degassing. The solution was subsequently dropped onto a polystyrene Petri dish lined with polyvinyl chloride (PVC) film as the substrate and spin-coated at 1000 rpm for 30 seconds, forming a uniformly thin film. Finally, the desired films were sequentially dried in an oven (Memmert UF55 universal laboratory and industrial heating oven) at 39 °C for 10 h.

**Figure 2:**
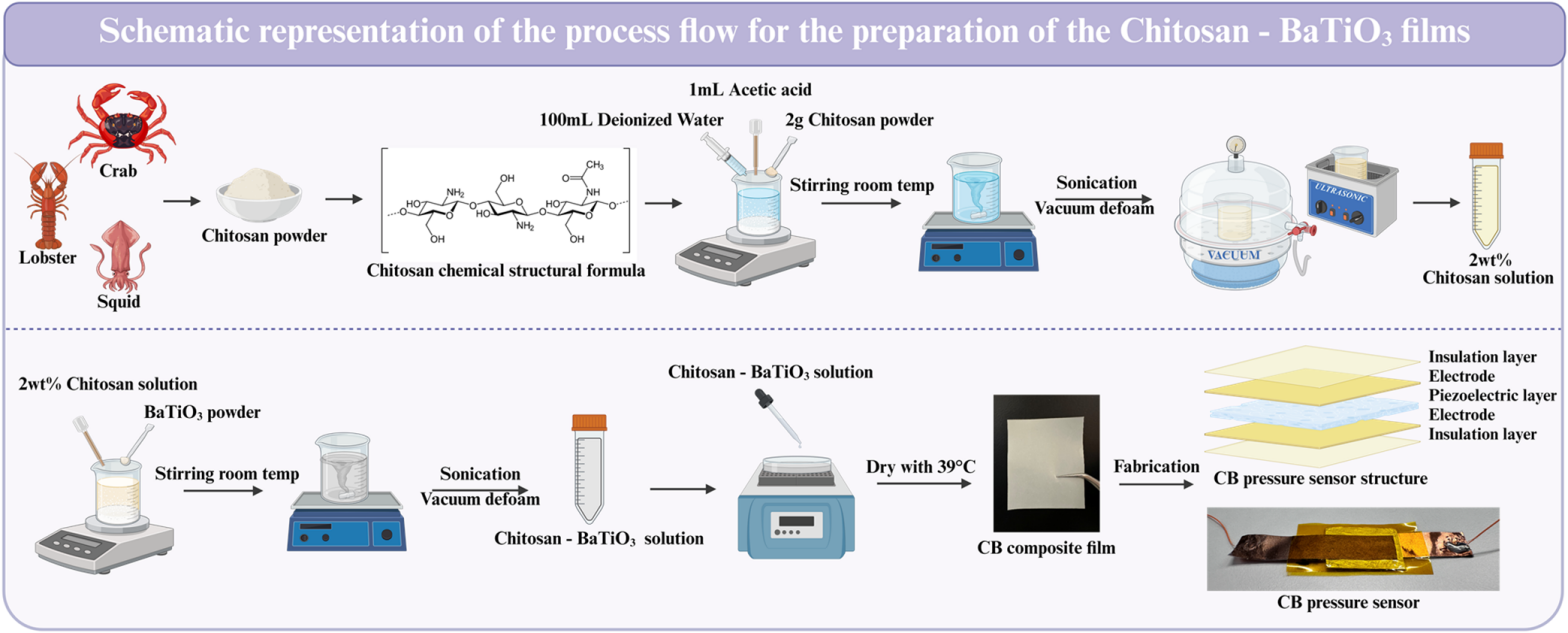
Schematic illustration of the fabrication process for CB composite films derived from marine biowaste and subsequent assembly into a CB pressure sensor device. Created in BioRender. Wang, Z. (2026) https://BioRender.com/ros21hg.

### Fabrication of the Chitosan-BaTiO_3_ Sensor

CB sensors were fabricated in a sandwich structure, comprising copper electrodes positioned on the top and bottom sides of the active piezoelectric layer. The active layers consisted of either pure chitosan, or 5, 10, or 15 wt % concentrations of CB composite films, each approximately 50 μm thick, with an active sensing area of 2.5 cm × 2.5 cm. For comparison, two pure chitosan films with different thicknesses were prepared as Chi-S1 (50 μm) and Chi-S2 (85 μm). All devices were encapsulated with polyimide tape, and all measurements were conducted under the same environmental conditions.

### Characterization and Testing

Surface morphology was characterized using field-emission scanning electron microscopy (SEM, FEI Nova Nano SEM). Crystalline phases were analyzed by X-ray diffraction (XRD, Rigaku MiniFlex 600, step size of 0.01°, scanning range from 10° to 70°2θ). Fourier-transform infrared spectroscopy (FTIR, Bruker Platinum A225) was utilized to examine the chemical structure. Film thickness measurements were conducted using a Logitech CG10 thickness gauge. Pressure sensitivity tests were performed by applying periodic compressive loads using a tunable-frequency vibration system (TV 50018, TIRA GmbH). Voltage outputs generated by the CB sensors were recorded with a digital storage oscilloscope (Keysight DSOX3014T). Mechanical testing was conducted using a stress–strain procedure, using an Instron 3367 universal testing machine.

### Statistical Methods

All the SEM images for the 3 different concentrations were obtained and are opened in the ImageJ software. Next, the images were converted to greyscale (8-bit) to obtain the histogram distribution for subsequent segmentation. The image contrast was increased to 0.6% saturated pixels and obtained clear distinguishable NPs from the polymer matrix, followed by the thresholding of NPs. Finally, the agglomeration fraction (AF) was calculated as the ratio of the total projected area of the agglomerates to the total analyzed image area (region of interest, ROI). The AF was determined using eq ([Disp-formula eq1])^31^

1 $$\mathrm{AF}=\frac{\sum {\mathrm{area}}_{\mathrm{agglomerates}}}{{\mathrm{area}}_{\mathrm{ROI}}}\times 100\%$$

## Results and Discussion

To explore the microstructural changes that occur during the process of fusing chitosan with different concentrations of BaTiO_3_ NPs, a range of analytical techniques were employed, including SEM, XRD and FTIR. Figure 3(a)–(c) shows images of the chitosan mixed with BaTiO_3_ NPs, and the bright spots represent the NPs in the chitosan polymer. At 5 wt %, the NPs in the chitosan are well dispersed and form a hydrogen bond with the NPs via the hydroxyl group (−OH) inside the chitosan, preventing them from sticking together as observed in Figure 3(a). As the concentration increased to 10 wt %, the NPs were very well uniformly distributed and were relatively distinct through moderate clusters as seen in Figure 3(b), whereas for the 15 wt %, the NPs were fully agglomerated with a very small separation distance between the NPs, clearly observed from Figure 3(c). These higher concentrations of the NPs make the composite film more brittle and less flexible. The grain size measured from SEM images using ImageJ (N = 50) gives average diameters of 0.12, 0.09, and 0.08 μm for the 5, 10, and 15 wt % BaTiO_3_ composites, respectively shown in Figure S1­(a)–(c), indicating a denser distribution of smaller nanoparticles with increasing BaTiO_3_ content.

**Figure 3:**
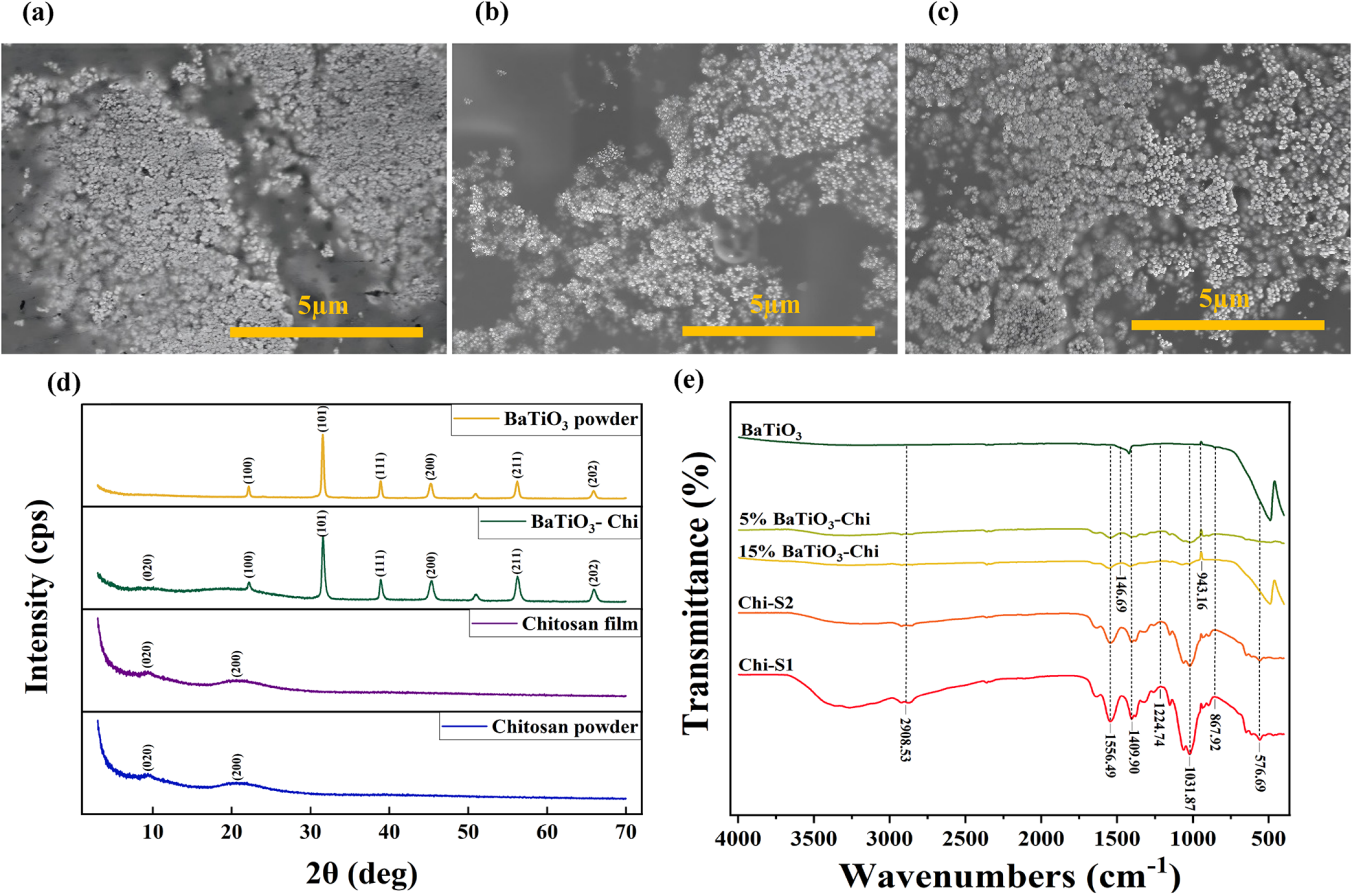
SEM image of CB composite film surface morphology under different concentrations (a) 5 wt %, (b) 10 wt % and (c) 15 wt %Grain size distribution histogram of the composite film. (d) XRD patterns for BaTiO_3_ powder, CB composite films, pure chitosan film, and chitosan powder. (e) FTIR spectra for BaTiO_3_ powder, CB composite films, and pure chitosan film Chi-S1 and Chi-S2.

The agglomeration fraction (AF) of BaTiO_3_ at different concentrations was further calculated as shown in Figure 3(a)–(c) using eq ([Disp-formula eq1]). The AF values for different concentrations (5, 10, 15 wt %) were obtained as the 45%, 54%, and 56%, respectively, and the obtained results suggest that the dispersion uniformity of BaTiO_3_ particles increases linearly with an increase in concentration of the BaTiO_3_. The crystalline structures of CB composite films were characterized by XRD as shown in Figure 3(d). The XRD pattern of BaTiO_3_ powder with distinct diffraction peaks at 2θ is determined as 22.3, 31.7, 39, 45, 51.1, 56.4, and 66° corresponding to the tetragonal phases (100), (101), (111), (200), (201), (211) and (202) respectively. Here, the highest peak appears at 31.7° (101). The peak intensity of the crystal plane (020) corresponding to the chitosan is low owing to the amorphous nature of the chitosan film, as compared to the dominating crystalline nature of the BaTiO_3_ NPs, clearly observed from the XRD of the composite film. FTIR analysis was performed to understand the intermolecular interactions between the chitosan and BaTiO_3_ NPs, where FTIR spectra with varying BaTiO_3_ content (0 wt %, 5 wt %,10 wt %,15 wt %) are shown in Figure 3(e). For pure chitosan film, a broad and intense band appears around 3430 cm^–1^, which is due to overlapping O–H and N–H stretching vibrations; followed by two distinct peaks appearing at 2921 cm^–1^ and 2908 cm^–1^ corresponding to asymmetric and symmetric C–H stretching vibrations, respectively. This confirms the sugar ring’s structural integrity. However, the intensity of these C–H vibrations gradually weakened with increasing BaTiO_3_ content, which may be due to physical dilution or to a weakening of chitosan’s contribution to the signals. Significant amide I (1655 cm^–1^, C=O stretching coupled to the C–N mode) and amide II (1556 cm^–1^, N–H bending) bands were observed, consistent with the characteristic functional groups of chitosan.

The output performance of CB sensors was systematically investigated under controlled mechanical stimulation using a tunable-frequency vibration system (TV 50018, TIRA GmbH). The results shown in Figure 4(a),4(b) summarize the output characteristics responding to pressure increasing from 20 to 80 kPa in 10 kPa increments and frequency increasing from 2 to 8 Hz in 1 Hz steps, respectively. A detailed comparison of the electrical output performance of CB sensors with different BaTiO_3_ concentrations is provided in Figure S2­(a),(b). All sensors exhibited a significant enhancement in output voltage with increasing pressure. The output voltage of Chi-S1 increased from 1.49 to 2.93 V. In comparison, the CB composites exhibited voltage enhancements from 1.01 to 7.6 V, 2.4 to 16.7 V, and 8.4 to 24.9 V, corresponding to BaTiO_3_ concentrations of 5, 10, and 15 wt %, respectively. As per the frequency response, all sensors show increased output voltage with increasing frequency and concentration of added BaTiO_3_ NPs. The 5 wt % CB composites demonstrated an output voltage range of 3.6 to 6.4 V, for 10 and 15 wt % CB composites showed broader response ranges, rising from 1.0 to 11.3 V and 7.9 to 23.3 V, respectively. The sensitivity for each CB composite sensor was then evaluated using OriginLab software to fit the experimental discrete data points linearly. Here, the slope is the sensitivity, and the variance can be quantified as the deviation between the fitted line and the actual values, as shown in Figure 4(**c**).

**Figure 4:**
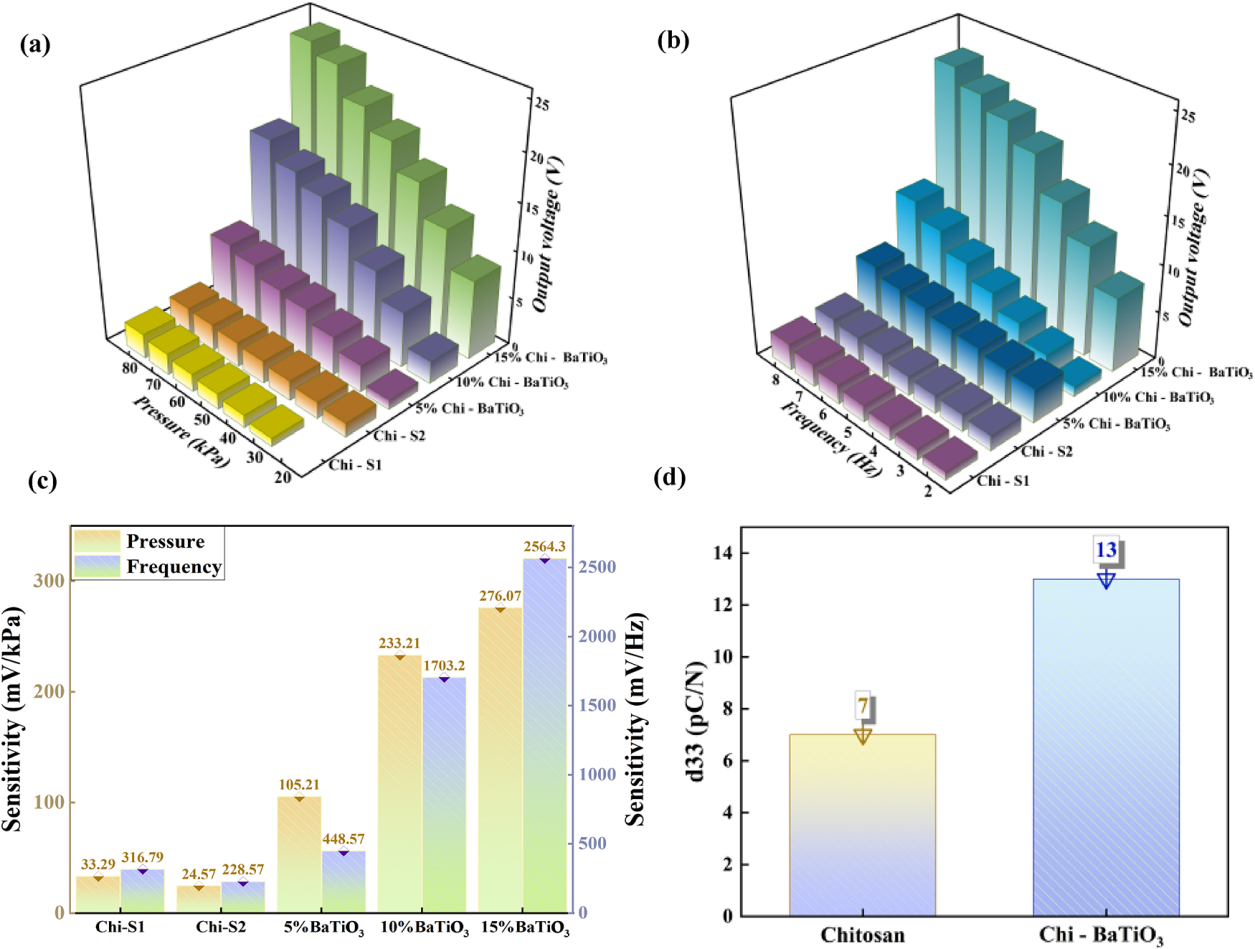
Output voltage response of CB sensors under varying pressures from 20 to 80 kPa in 10 kPa increments at a fixed frequency of 8 Hz. (b) Output voltage response under varying frequencies from 2 to 8 Hz in 1 Hz increments at a fixed pressure of 80 kPa. (c) Pressure and frequency sensitivity comparison among chitosan films and CB composites. (d) Piezoelectric coefficient (*d*_33_) comparison between pure chitosan and 15 wt % CB composites.

These results indicate that pressure sensitivity (*S*_*p*_) is significantly improved by adding 5 wt % BaTiO_3_, where the measured response rises from 24 mV/kPa to 105 mV/kPa, followed by an increase of 233 mV/kPa to 276 mV/kPa, corresponding to 10 and 15 wt % BaTiO_3,_ respectively as shown in Figure S3­(a)–(e). The variance of all fits is close to 1, indicating good sensor linearity in the 10–80 kPa range. The frequency sensitivities were also obtained using the same treatment as presented in Figure S3­(f)–(j). The frequency sensitivity exhibited for pure chitosan film was 228 mV/Hz, and after adding BaTiO_3_, the frequency sensitivity (*S*_*f*_) was 448 mV/Hz, 1703 mV/Hz, and 2564 mV/Hz, corresponding to contents of 5, 10, and 15 wt % BaTiO_3_, respectively. It is evident that with the doping of BaTiO_3_, the sensitivity of the CB sensor has been significantly improved due to the enhanced *d*_33_ for 15 wt % CB composites, which can better meet the practical requirements. A quasi-static test is done by applying a force of 0.25 N at a frequency of 110 Hz to obtain the *d*_33_ value. This enhancement can be attributed to the following mechanism. The FTIR spectrum confirms that only physical interactions occurred during the incorporation of BaTiO_3_ nanoparticles into chitosan, without the breaking or formation of any chemical bonds. Although the agglomeration fraction increased from 45 to 56% with rising BaTiO_3_ nanoparticle concentration, the particle diameter analysis and SEM images indicate that the nanoparticles are more uniformly dispersed within the chitosan matrix at smaller individual diameters, while remaining uniformly and fully encapsulated by the chitosan polymer, with no large agglomerated clusters present. Locally high-filling regions formed by appropriately dispersed BaTiO_3_ nanoparticles generate numerous interfacial electric field hotspots, which facilitate enhanced local electric field polarization when the film is subjected to external pressure, thereby increasing the effective piezoelectric coefficient and leading to higher pressure sensitivity. Well-dispersed individual nanoparticles thus contribute effectively to the piezoelectric response under mechanical loading. In contrast, nanoparticles in direct contact within large agglomerated clusters experience internal slippage under applied pressure, interrupting the stress transfer pathway and preventing the generation of effective piezoelectric charges.^17^ A similar trend has been validated by Ciomaga et al. in BaTiO_3_ nanocube and gelatin composite films, where the piezoelectric coefficient *d*_33_ increased from 7 pm/V in pure gelatin to 21 pm/V at 40 wt % BaTiO_3_ loading.^32^ As shown in Figure 4(d), the *d*_33_ increased significantly from 7 pC/N for pure chitosan to approximately 13 pC/N after incorporating BaTiO_3_, indicating enhanced piezoelectric properties. Additionally, response time is a key performance parameter for evaluating sensors. Here, the response time is defined as the 10–90% rise from the baseline to the steady-state amplitude, and the recovery time as the 90–10% decay back to the baseline. As shown in Figure 5(a), the CB sensor exhibits a response time of 0.24 ms and a recovery time of 0.64 ms, demonstrating ultrafast dynamics and corroborating its high sensitivity. Another key performance parameter, hysteresis, refers to the difference in the output voltage of the sensor between the loading and unloading paths under the same applied pressure, which reflects the measurement accuracy of the sensor and the energy dissipation during the deformation process.^33^ As shown in Figure S4, stepped compressive loading was applied to the 15 wt % CB composite sensor. The pressure was sequentially increased from 20 to 80 kPa and then decreased back from 80 to 20 kPa. The output voltage curves recorded in the forward and reverse directions demonstrate similar pressure sensitivities, with the maximum output voltage difference of 0.8 V observed at 50 kPa, and the full-scale output voltage span across the measurement range was 16.5 V. The calculated hysteresis of the CB pressure sensor is 4.85% according to eq ([Disp-formula eq2]),^34^ expressed as

2 $$H_{\mathrm{full}\;\mathrm{scale}}\;\%=\frac{\max\left|V_{\mathrm{loading}}-V_{\mathrm{unloading}}\right|}{V_{\mathrm{full}\;\mathrm{scale}}}\times 100\%$$

**Figure 5:**
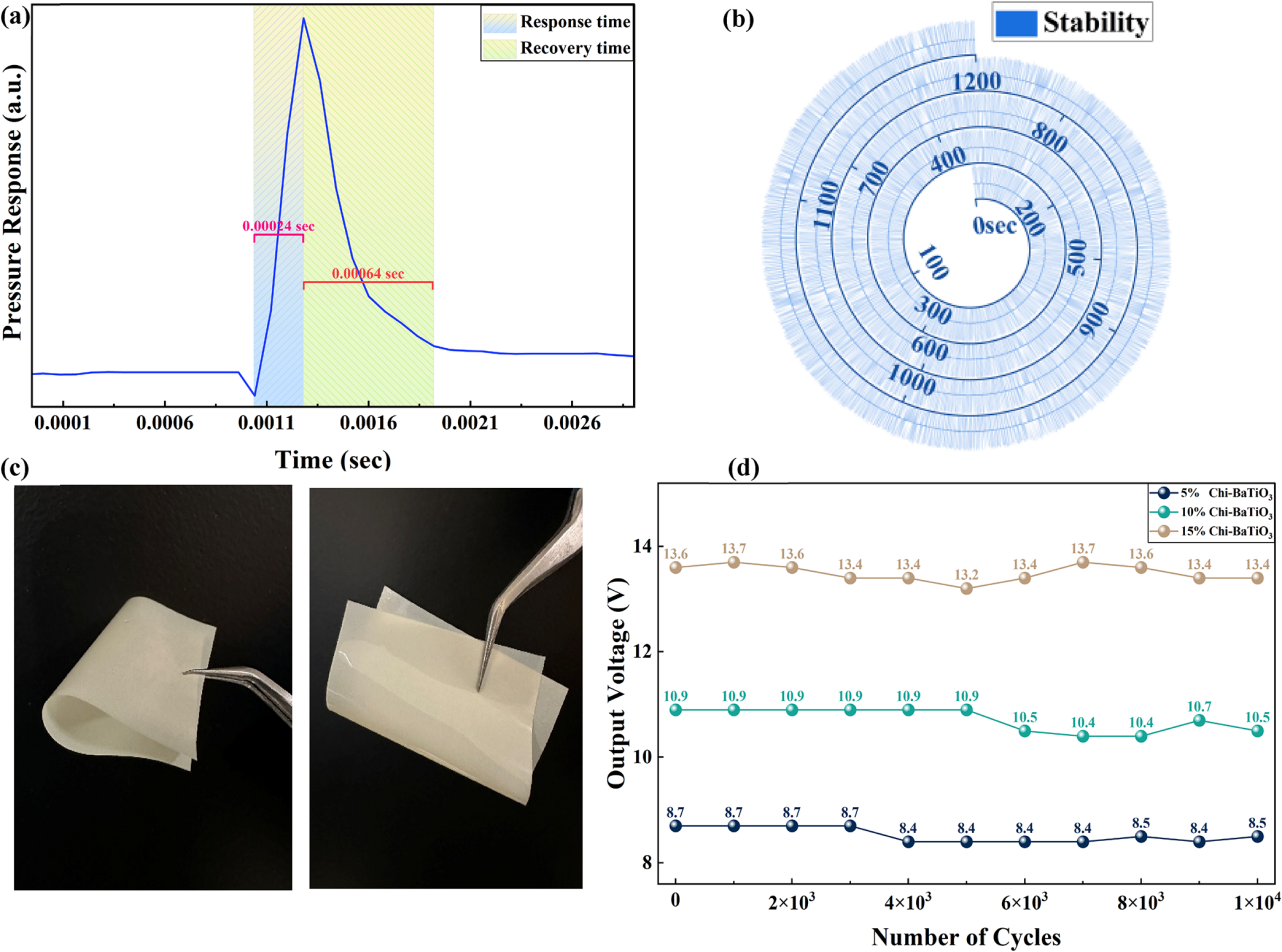
Transient pressure response of the CB sensor, showing a response time of 0.24 ms and a recovery time of 0.64 ms under a single pressure impulse. (b) Long-term stability under continuous cyclic loading, illustrated over 1200s with sustained, repeatable applied pressure. Bending reliability tests of the CB sensor: (c) CB film under bending deformation and (d) output-voltage stability after 10^4^ bending cycles for different BaTiO_3_ concentration (5, 10, and 15 wt %).

This hysteresis value is comparable to those reported for flexible piezoelectric pressure sensors with soft polymeric matrices, suggesting that the CB sensor exhibits reliable response under repeated mechanical loading suitable for dynamic sensing applications. Figure 5(b) illustrates the loading stability of the CB sensor over 10000 cycles, showing a consistent voltage output and confirming its high levels of durability and reliability under repeated mechanical loading. To further assess its suitability for wearable applications, additional cyclic bending tests were performed shown in Figure S5­(a)–(c), and the detailed results are provided in Figure 5(c),(d). The sensor maintained stable output after 10^4^ bending cycles, demonstrating good bending durability under repeated deformation. Moreover, the device was fully encapsulated with PI film to minimize the influence of external environmental factors such as humidity, sweat, and water exposure during operation. A stress–strain analysis on chitosan and its composite film was performed. Figure 6(a),(b) show images of the CB films attached to the Instron machine and stress as a function of strain, respectively. The obtained force displacement curves show that the relative stiffness of the 15 wt % composite exhibits higher stiffness as compared to those of the lower concentrations. The Young’s modulus of elasticity was calculated from the linear region of the force–displacement curves as per eq ([Disp-formula eq3])^15,35^

3 $$E=\frac{dF}{d\Delta L}\;\frac{L_{0}}{A}$$

where *L*_0_ is the initial length of the gauge as 40 mm, *A* is the area of the cross-section, and $\frac{dF}{d\Delta L}$ is the slope of the elastic region. The tensile modulus for the CB materials of different concentrations across 5, 10, and 15 wt %, was obtained as 6.21 GPa, 9.14, and 10.53 GPa, respectively. The results suggest that increasing the BaTiO_3_ concentration improved the rigidity of the composite films, possibly due to enhanced load transfer between the NPs and the chitosan matrix.^36^ The biocompatibility and degradability of chitosan and its composites are very well reported.^37–39^ In this research, a further biodegradability test on the chitosan and its mass loss rate were performed, as represented in Figure 7(a),(b) respectively. The mass of chitosan decreased to 7% within 25 min and was almost completely degraded after 45 min. This rapid degradation behavior confirms the biodegradable nature of chitosan in aqueous environments while also supporting the growing demand for sustainable, biodegradable, and recyclable material systems in green electronics. Next, real-time tests were performed on the pressure sensor, and in-sole experimental measurements were done. The obtained results from the pressure sensor for different persons of varying weights are shown in Figure 8(a)–(c), and it can be clearly seen that the output voltage varies linearly with an increase in the weight of the person, demonstrating good linear sensitivity of the as-fabricated pressure sensor.

**Figure 6:**
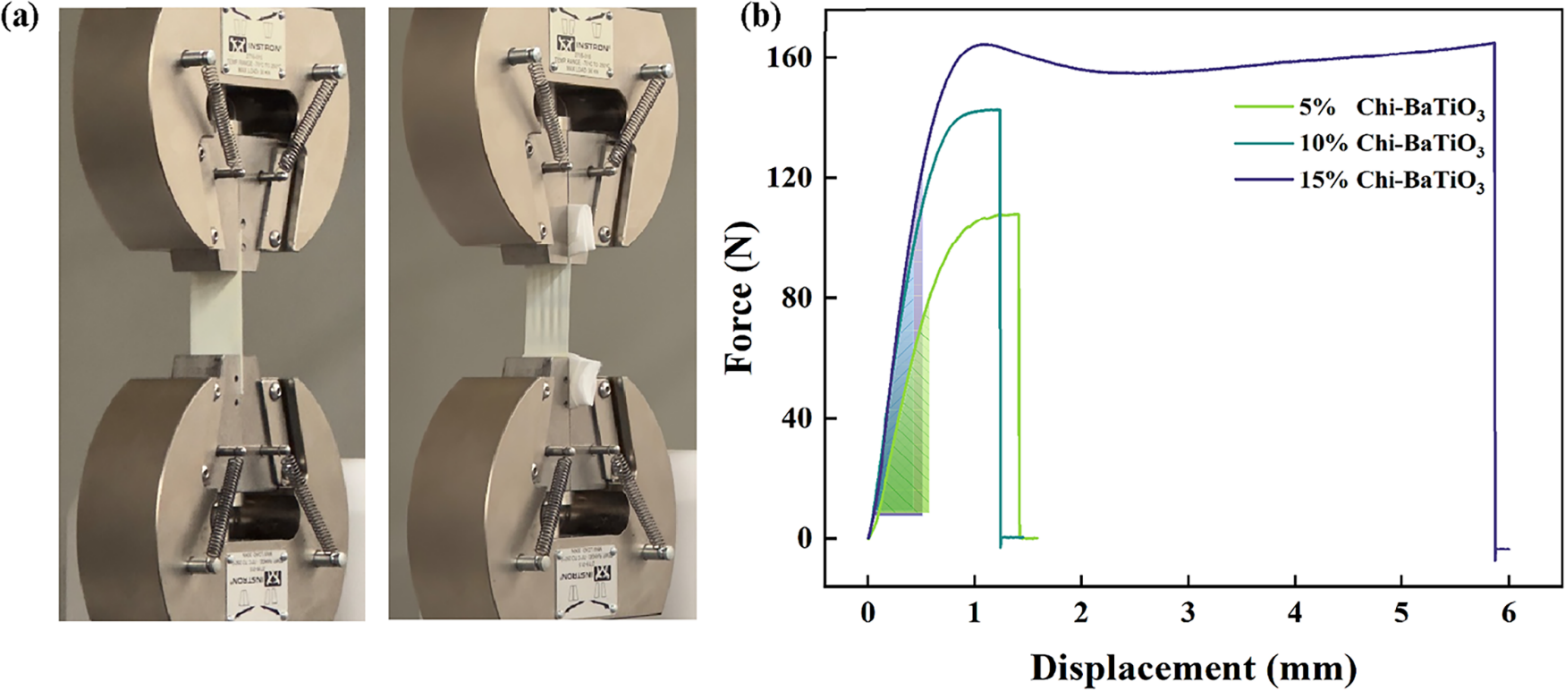
Stress–strain analysis of CB (a) Instron test setup with the film specimen mounted in the grips, and (b) compressive stress–strain curves of CB films with different BaTiO_3_ loadings (5, 10, and 15 wt %).

**Figure 7:**
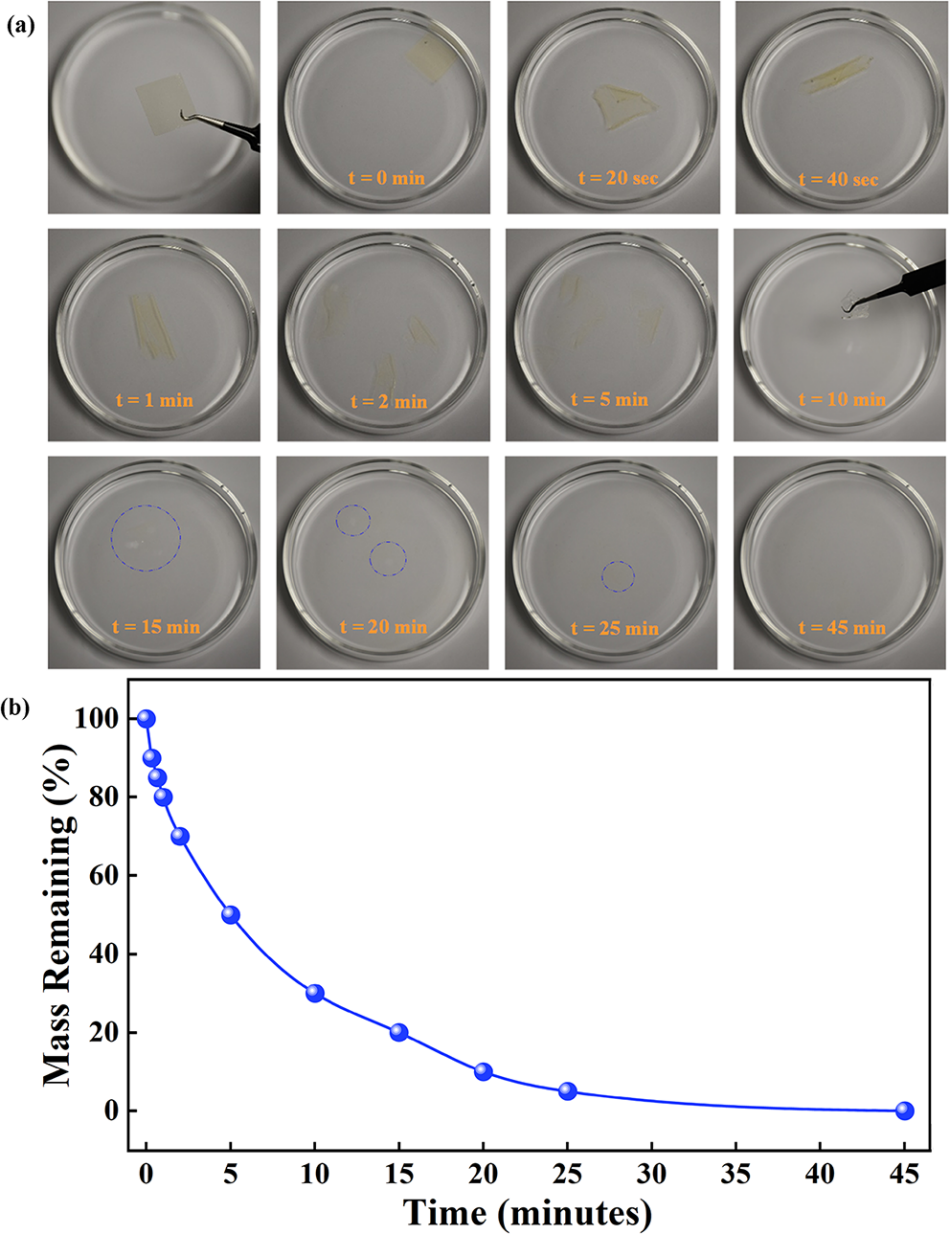
Biodegradability investigation of pristine chitosan films in deionized water (pH 6.5): (a) time-dependent dissolution of the chitosan film during immersion, and (b) the corresponding mass-loss rate (weight change) over time.

**Figure 8:**
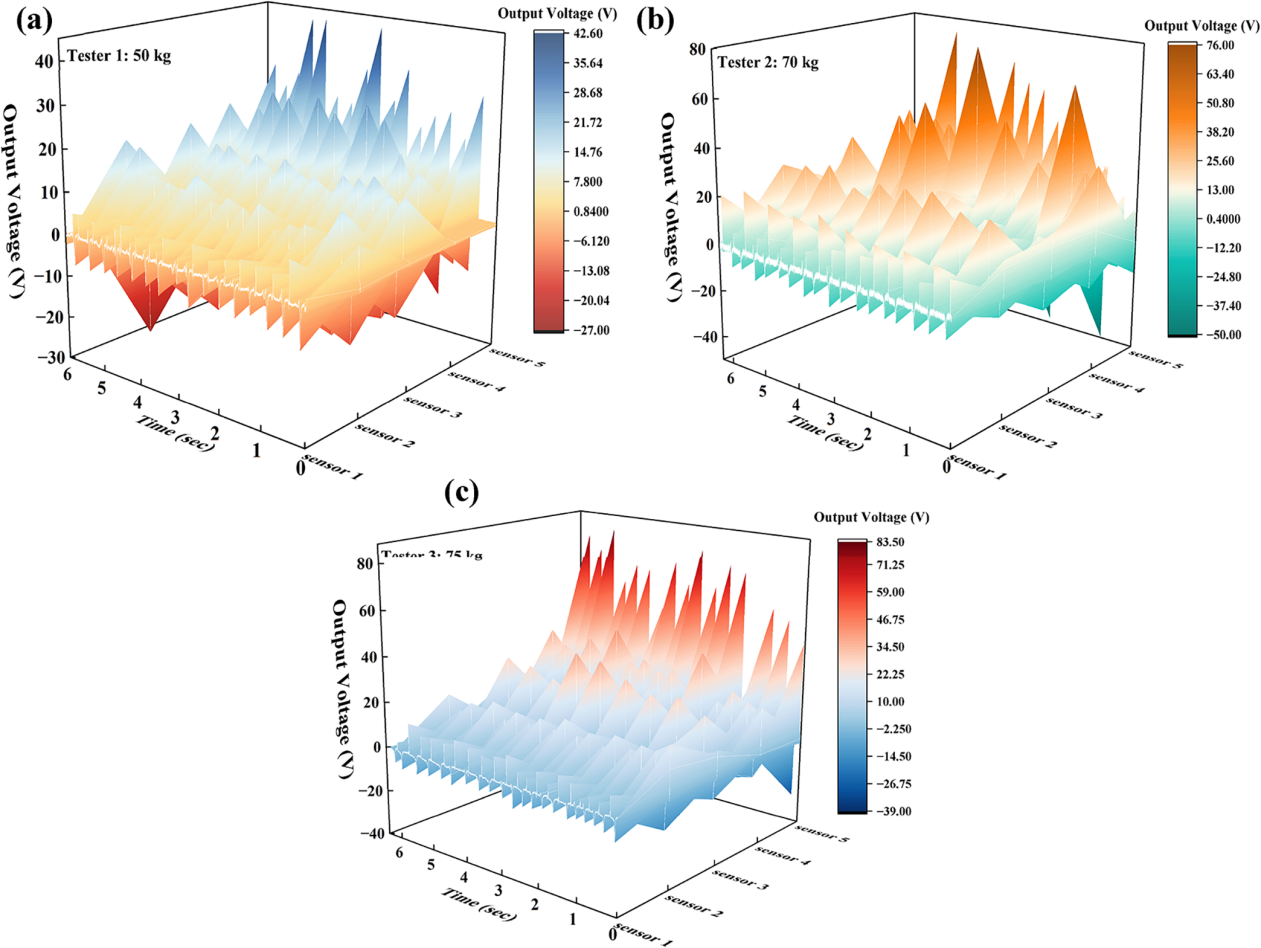
Shows the output-voltage distribution of the CB sensor array recorded from participants with different body weights: (a) Tester 1 (50 kg), (b) Tester 2 (70 kg), and (c) Tester 3 (75 kg).

Table 1 demonstrates the performance of pressure sensors fabricated from different materials in terms of sensitivity, output voltage, piezoelectric coupling coefficient, and biocompatibility. The pressure sensor manufactured from the CB composite material in this study exhibits a significantly higher sensitivity value than other materials. Among these, Glycine/PVA exhibits a frequency sensitivity of 50 mV/Hz, whereas pure PVDF film exhibits a comparatively low 8 mV/kPa pressure sensitivity. Although PVDF and its composites possess high piezoelectric coupling coefficients, they are unsuitable for biomedical applications due to inadequate biocompatibility. Materials with exceptional biocompatibility, such as Parylene C, Glycine, and Chitosan, exhibit lower *d*_33_ coefficients. However, the Chitosan-BaTiO_3_ composite material presented herein exhibits a higher *d*_33_ value compared to other biocompatible materials, coupled with greater voltage output and shorter response time. In summary, CB films demonstrate superior advantages in sensitivity and response time, making them more suitable for practical applications, including for wearable healthcare technologies.

**Table 1:** Performance Comparison of Various Piezoelectric Sensors, Highlighting the Superior Sensitivity of the CB Sensor

material	sensitivity	output voltage	*d*_33_	response time	biocompatibility	refs
PVDF	8 mV/kPa	1.6 V	30.79 pC/N	55 ms	NO	^40,41^
PVDF-ZnO	33 mV/kPa	11 V	150 pC/N	16 ms	NO	^42,43^
Parylene C	87.62 mV/kPa	9.6 V	1.8 pC/N	12 ms	YES	^13^
glycine/PVA	50 mV/Hz	4.1 V	5.3 pC/N		YES	^44^
glycine/chitosan	2.87 mV/kPa	190 mV		<100 ms	YES	^37^
chitosan/BaTiO_3_	276 mV/kPa	24.9 V	13 pC/N	0.24 ms	YES	this work
	2564 mV/Hz	23.3 V				

## Conclusion

In this research, a highly sensitive, self-powered pressure sensor was fabricated using a hybrid chitosan-BaTiO_3_ NPs composite. The pressure sensitivity of the sensor was enhanced from 24.5 mV/kPa to 276 mV/kPa by adding 15 wt % BaTiO_3_, and the frequency sensitivity improved from 228 mV/Hz to 2564 mV/Hz. Furthermore, the nontoxic, biocompatible and flexible nature of this new composite material suggests that it has considerable potential for a wide range of applications in the medical and wearable devices field.

## Supplementary Material



## Notes

**Figure** **S1**: SEM images of grain-size distribution histogram of the CB composite film under different concentrations (a) 5 wt %, (b) 10 wt %, and (c) 15 wt %; **Figure** **S2**: Electrical output performance of CB sensors with different BaTiO_3_ concentrations under an applied force of 5 N at 5 Hz: (a) output voltage and (b) output current; **Figure** **S3**: Plots of piezoelectric sensitivity for pure chitosan and CB sensors versus applied pressure (**a)**–**(e)** and frequency **(f)**–**(j)**. (a) Output voltage vs pressure for pure chitosan-S1, with a sensitivity of 33.29 mV/kPa. (b) Pure chitosan-S2, 24.57 mV/kPa. **(c) - (e)** CB composites with 5%, 10%, and 15% contents of BaTiO_3_, giving 105.21, 233.21, and 276.07 mV/kPa, respectively. (f) Frequency response of pure chitosan-S1, 316.79 mV/Hz. (g) Pure chitosan-S2, 228.57 mV/Hz. **(h)**–**(j)** CB composites with 5%, 10%, and 15% contents of BaTiO_3_, yielding 448.57, 1703.21, and 2564.29 mV/Hz, respectively; **Figure** **S4**: Hysteresis characterization of the 15 wt % CB composite pressure sensor under stepwise compressive loading and unloading between 20 and 80 kPa at a fixed frequency of 8 Hz; and **Figure S5:** Bending reliability tests of the CB sensor: **(a**_**1**_ **)**–**(a**_**3**_ **)** CB film under bending deformation, (b) 3D printed modules for the bending-tests showing multiple controlled angles (0–180°) (PDF)

## Notes

James Watt School of Engineering, University of Glasgow, University Avenue, Glasgow G12 8QQ, U.K

## Abbreviations

IoT: Internet of Things
NPs: nanoparticles
SEM: scanning electron microscopy
XRD: X-ray diffraction
FTIR: Fourier transform infrared spectroscopy
wt %: weight percent
Sp: pressure sensitivity
Sf: frequency sensitivity
EPSRC: Engineering and Physical Sciences Research Council
PZT: lead zirconate titanate
PVDF: polyvinylidene fluoride
PDLA: poly­(D-lactic acid)
PLA: polylactic acid
P­(VDF-TrFE): poly­(vinylidene fluoride-trifluoroethylene)
PA: polyamides
CB: chitosan–BaTiO_3_
PVC: polyvinyl chloride
DI: deionized (water)
FEI: field-emission instruments
pC/N: picocoulombs per newton
PVA: poly­(vinyl alcohol)
