# Supplementary material for: A Hybrid Chitosan–BaTiO3 Composite-Based Flexible and Self-Powered Pressure Sensor for Wearable Healthcare Applications
Source: ACS Appl Eng Mater. 2026 Jun 17;4(7):3620–30. doi: 10.1021/acsaenm.6c00330 (PMC13411042; doi:10.1021/acsaenm.6c00330)
Supplement: Supplementary file 1 [file em6c00330_si_001.pdf]

Supporting Information

on

A hybrid Chitosan-$BaTiO_3$ composite-based flexible and self-powered pressure sensor for wearable healthcare applications

Zhao Wang[1], Bhavani Prasad Yalagala[2], Jungang Zhang[2], Zixuan He[3], Hadi Heidari[1,2], Andrew Feeney[1,3]*

[1.] Centre for Medical and Industrial Ultrasonics, James Watt School of Engineering, University of Glasgow, University Avenue, Glasgow, G12 8QQ, United Kingdom

[2.] Microelectronics Laboratory, James Watt School of Engineering, University of Glasgow, Glasgow, University Avenue, Glasgow, G12 8QQ, United Kingdom

[3.] Materials and Manufacturing Research Group, James Watt School of Engineering, University of Glasgow, University Avenue, Glasgow, G12 8QQ, United Kingdom

E-mail: Andrew.Feeney@glasgow.ac.uk

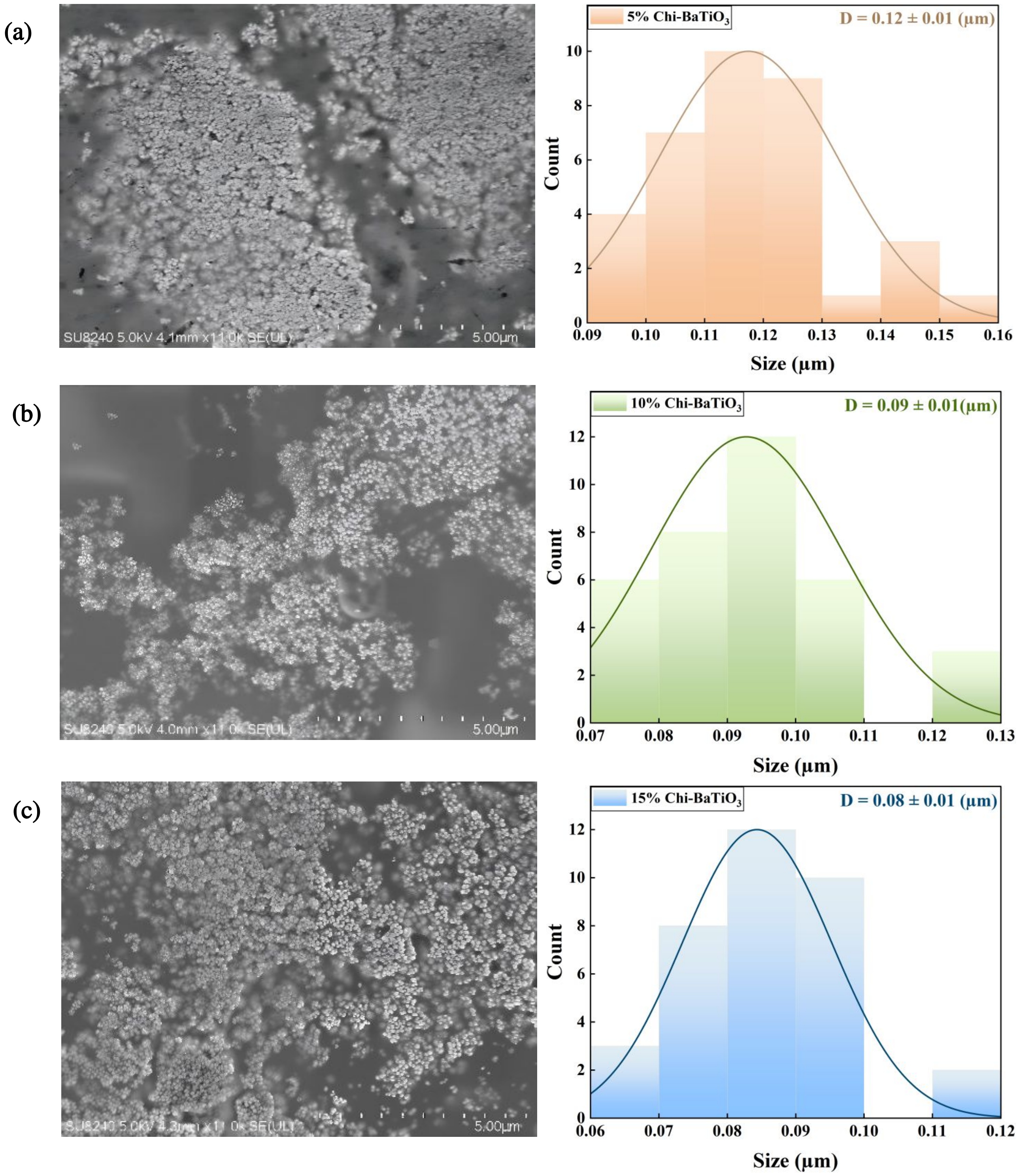

Figure. S1. SEM images of grain size distribution histogram of the CB composite film under different concentrations (a) 5 wt.% (b) 10wt.% and (c) 15 wt.%.

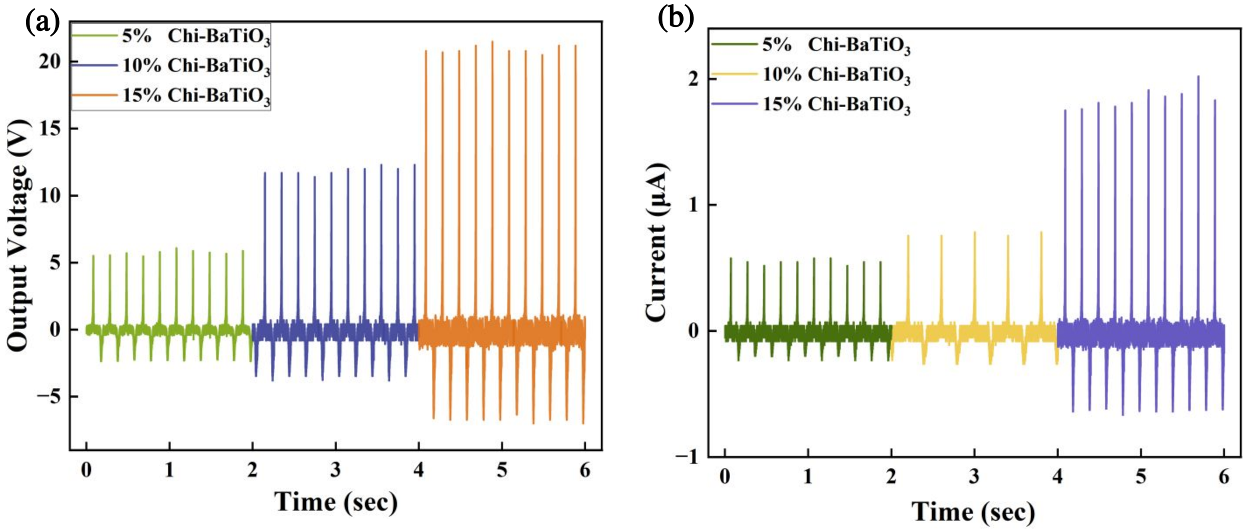

Figure. S2. Electrical output performance of CB sensors with different $BaTiO_3$ concentrations under an applied force of 5 N at 5 Hz: (a) output voltage and (b) output current.

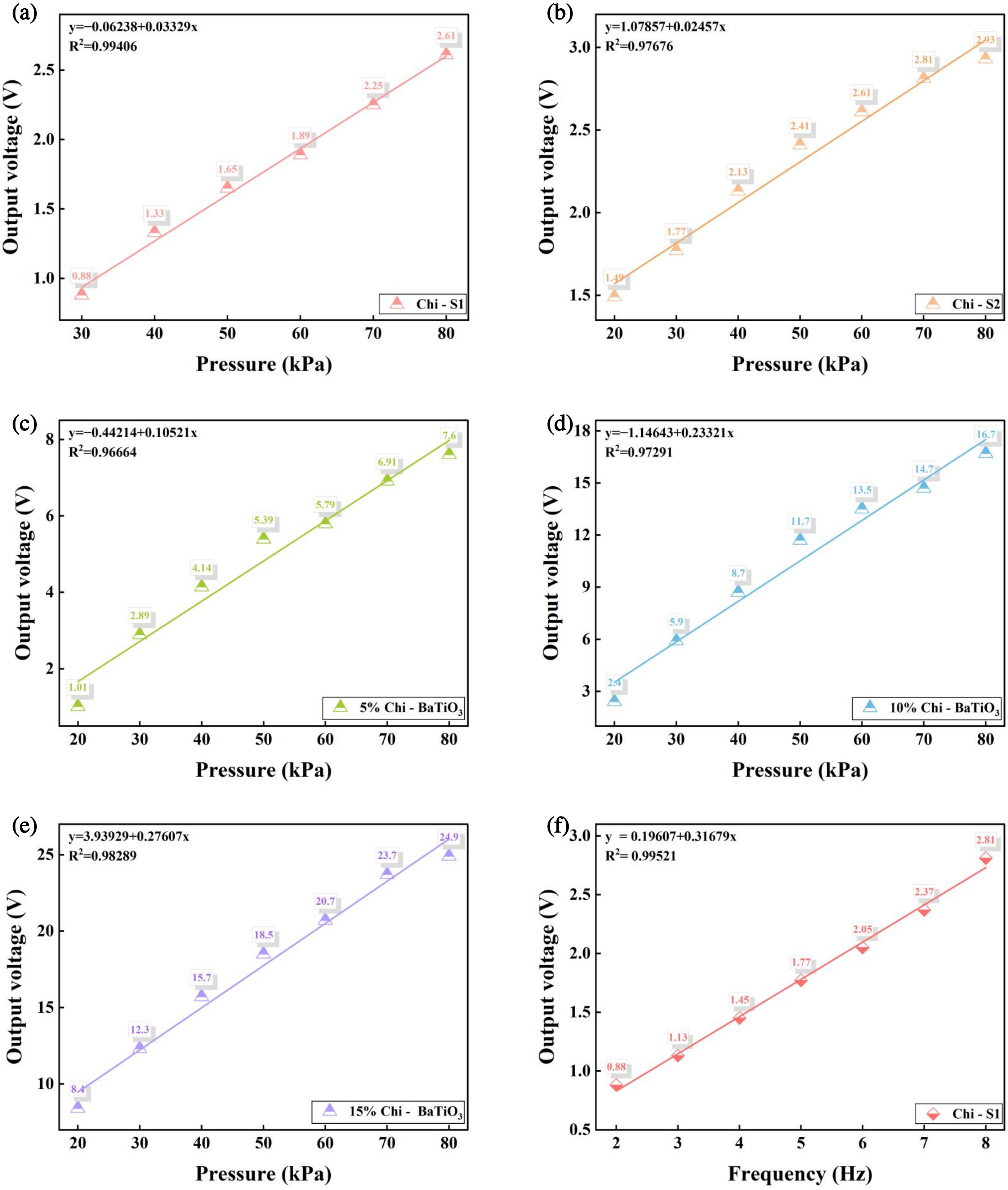

(a)
y=−0.06238+0.03329x
R2=0.99406
Output voltage (V)
Pressure (kPa)
Chi - S1
0.88
1.33
1.65
1.89
2.25
2.61
(b)
y=1.07857+0.02457x
R2=0.97676
Output voltage (V)
Pressure (kPa)
Chi - S2
1.49
1.77
2.13
2.41
2.61
2.81
2.93
(c)
y=−0.44214+0.10521x
R2=0.96664
Output voltage (V)
Pressure (kPa)
5% Chi - BaTiO3
1.01
2.89
4.14
5.39
5.79
6.91
7.6
(d)
y=−1.14643+0.23321x
R2=0.97291
Output voltage (V)
Pressure (kPa)
10% Chi - BaTiO3
2.4
5.9
8.7
11.7
13.5
14.7
16.7
(e)
y=3.93929+0.27607x
R2=0.98289
Output voltage (V)
Pressure (kPa)
15% Chi - BaTiO3
8.4
12.3
15.7
18.5
20.7
23.7
24.9
(f)
y = 0.19607+0.31679x
R2= 0.99521
Output voltage (V)
Frequency (Hz)
Chi - S1
0.88
1.13
1.45
1.77
2.05
2.37
2.81

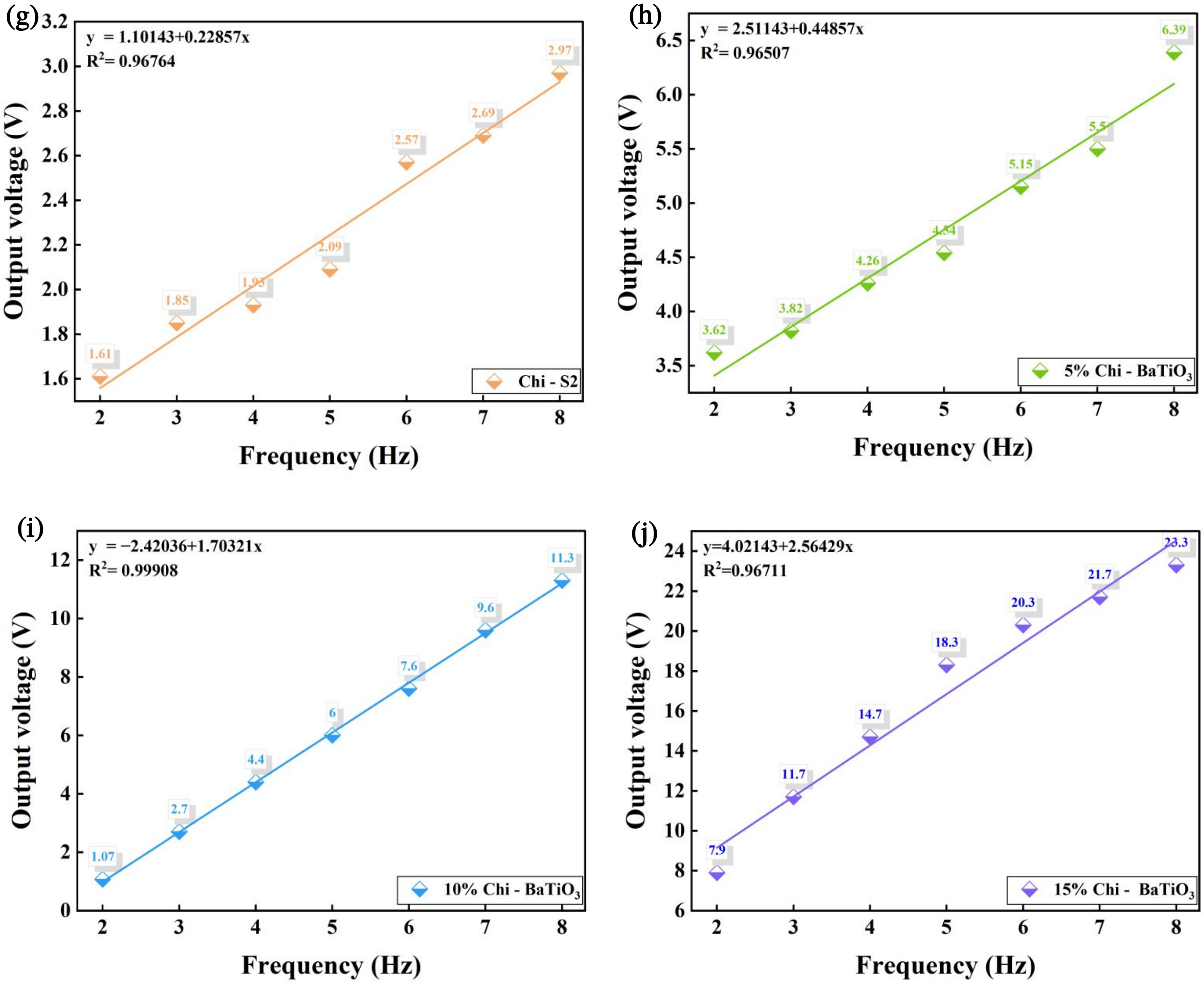

Figure. S3. Plots of piezoelectric sensitivity for pure chitosan and CB sensors versus applied pressure (**a) - (e)** and frequency **(f) - (j)**. **(a)** Output voltage vs. pressure for pure chitosan-S1, with a sensitivity of 33.29 mV/kPa. **(b)** Pure chitosan-S2, 24.57 mV/kPa. **(c) - (e)** CB composites with 5%, 10%, and 15% contents of $BaTiO_3$, giving 105.21, 233.21, and 276.07 mV/kPa, respectively. **(f)** Frequency response of pure chitosan-S1, 316.79 mV/Hz. **(g)** Pure chitosan-S2, 228.57 mV/Hz. **(h) – (j)** CB composites with 5%, 10%, and 15% contents of $BaTiO_3$, yielding 448.57, 1703.21, and 2564.29 mV/Hz, respectively.

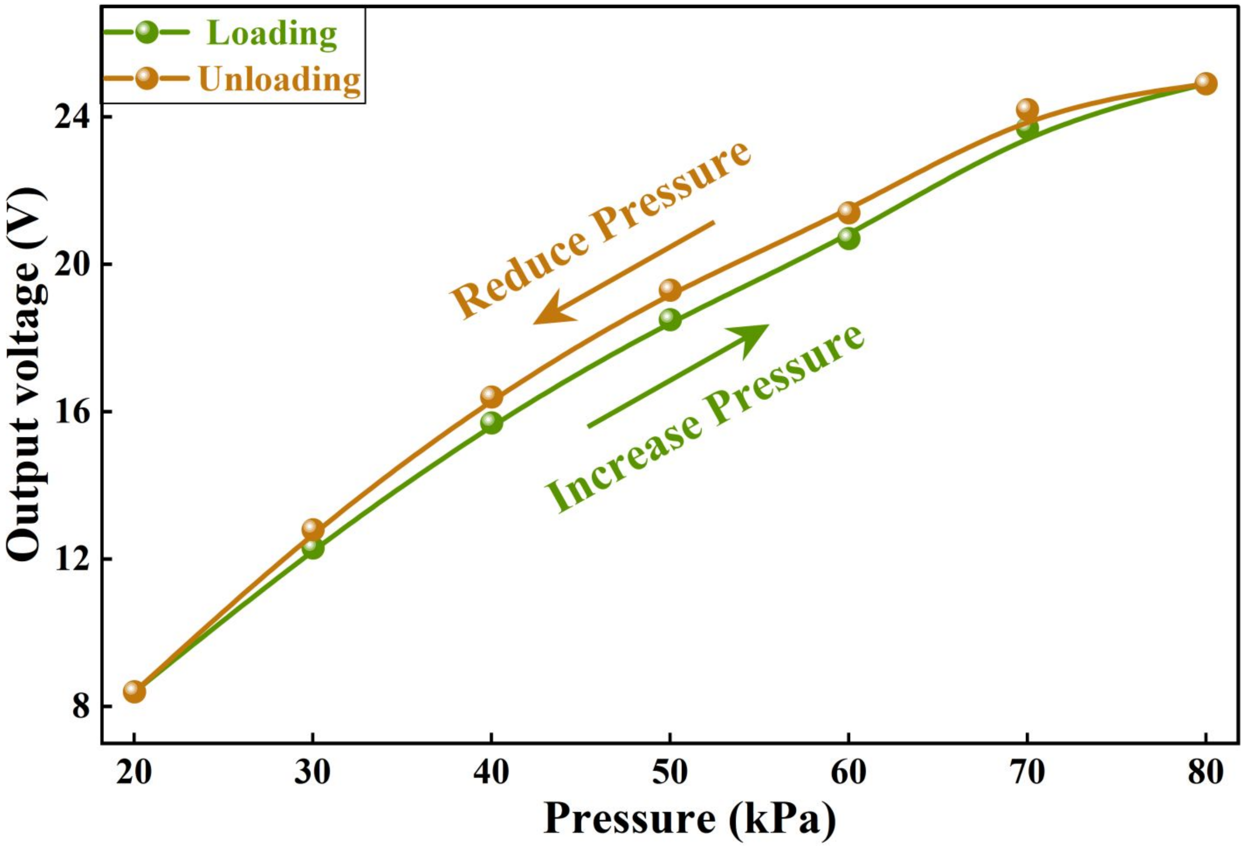

Figure. S4. Hysteresis characterisation of the 15 wt.% CB composite pressure sensor under stepwise compressive loading and unloading between 20 and 80 kPa at a fixed frequency of 8 Hz.

(a$_1$) (a$_2$) (a$_3$)

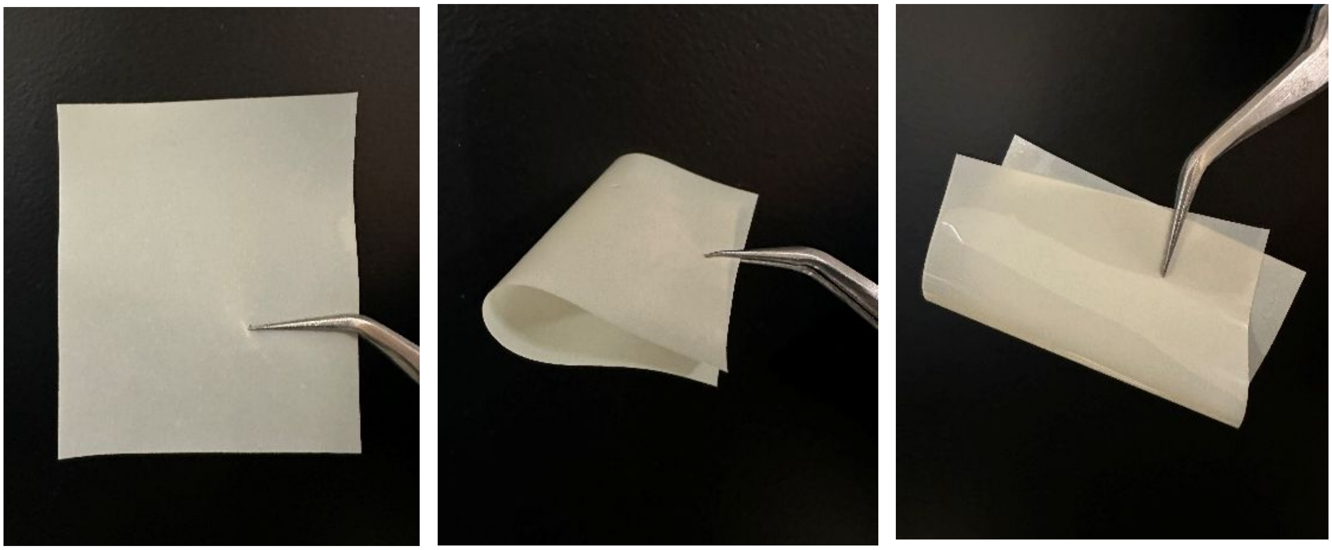

(b)

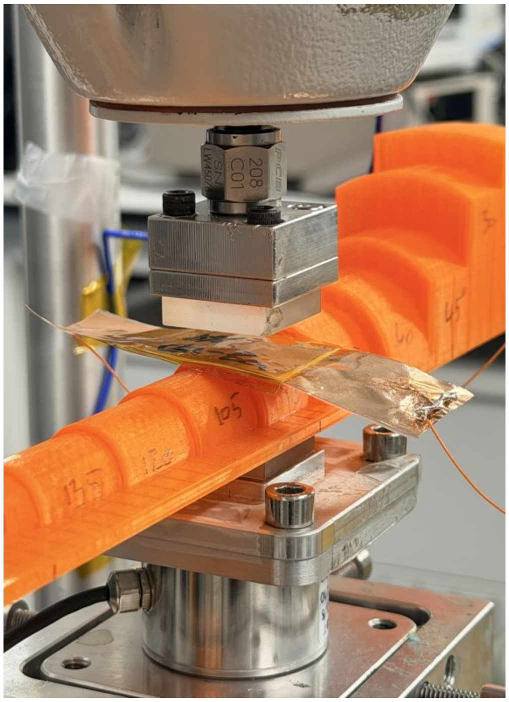

Figure. S5. Bending reliability tests of the CB sensor: **(a$_1$)-(a$_3$)** CB film under bending deformation, **(b)** 3D printed modules for the bending-tests showing multiple controlled angles (0–180°).
